# The Effect of the Return of Serve on the Server Pair’s Movement Parameters and Rally Outcome in Padel Using Cluster Analysis

**DOI:** 10.3389/fpsyg.2019.01194

**Published:** 2019-05-28

**Authors:** Jesus Ramón-Llin, Jose Francisco Guzmán, Salvador Llana, Rafa Martínez-Gallego, Nic James, Goran Vučković

**Affiliations:** ^1^Faculty of Teaching, University of Valencia, Valencia, Spain; ^2^Faculty of Physical Activity and Sport, University of Valencia, Valencia, Spain; ^3^Faculty of Science and Technology, London Sport Institute, Middlesex University, London, United Kingdom; ^4^Faculty of Sport, University of Ljubljana, Ljubljana, Slovenia

**Keywords:** cluster analysis, performance analysis, padel, return of serve, movement parameters

## Abstract

**Purpose:** The pressure exerted on racket sports players by the service has been well documented. Whilst the return of serve has been suggested through qualitative interviews as being of similar importance there is a dearth of quantitative data to support this contention. This study analyzed time, speed, and distance parameters related to the outcome of the return of serve (ROS) in Padel, a sport similar to tennis but played on a court bounded by walls and played in doubles format only.

**Methods:** Matches (*n* = 18) at two tournaments, sanctioned by the Valencian Federation, in 2012 were recorded and processed using Tracker software. ROS shot type (flat or lob), ball location, players’ positions on court and movement parameters between the ROS and the third shot of the server were captured 25 times per second.

**Results:** Both lob and flat ROS produced six main clusters, as well as a small proportion of shots deemed outliers. The clusters differentiated shots played by two different level players (National and Regional), whether the ROS was played following a first or second serve, whether the serving pair adopted a conventional or Australian formation and whether the rally ended in a short number of shots (seven or less) or not.

**Conclusion:** It was suggested that the aim of the ROS in Padel was to prevent the serving pair winning the rally quickly, since the advantage of the serve diminished after around 6 to 8 shots. This was best achieved by good depth on lobs, regardless of the direction, and pace on low shots, predominately aimed toward the server. This approach should be further modified to include the time between serve and ROS and consideration could be given to classifying attacking and defending positions.

## Introduction

Padel is a complex and dynamic racket sport, played by two players as a pair (doubles only) on a court (10 × 20 m), comparable to tennis, but bounded by walls, a fence and an opening next to the net. Matches are the best of three sets using tennis scoring. At the elite level, average playing time for male players is just under 1 h with ball in play time approximately 35% of the total time (Torres-Luque et al., 2015). Castillo-Rodríguez et al. (2014) found that players of different standard averaged between 609 and 1043 m per set, but higher ranked players covered less distance than lower ranked players. However, in closely contested elite level matches, players covered 1470 m during ball in play time (Ramón-Llin et al., 2014).

As with other racket sports, Padel players tend to apply tactics depending on the situation, either advantageous or disadvantageous, which determines shot selection typically based on players’ movement or positioning. Tactical analysis has showed that Spanish players at National and Regional levels used more volleys, trays and smashes (National 28.3%, 8.9%, 5%; Regional 26.2%, 11.8%, 4.1%, respectively) than amateur players (16.7%, 7.4%, 2.9%; Ramón-Llin et al., 2017). These results indicated different tactical behaviors, for players of different standard, which also implied different positioning by players, i.e., greater net dominance allowing the increase in these attacking shots. The importance and effectiveness of players’ tactical positioning has also been analyzed in squash and tennis. For example, Vučković et al. (2008, 2009) found that the frequency of occupying the T area in squash, at the moment the opponent played their shot, best discriminated playing standard. Winners of a game also spent a greater proportion of total playing time on the T, suggested as indicative of a player’s dominance. In tennis, Martinez-Gallego et al. (2013) showed that game winners spent less time in defensive zones than losers suggesting that successful performance was related to offensive tactics. However, gender and court surface have been shown to have an effect on tactical parameters during tennis Grand Slam tournaments (O’Donoghue and Ingram, 2001). Offensive strategy was assessed in elite Padel, finding that 60% of points were either won or lost at the net (Courel-Ibáñez et al., 2015). The authors concluded that being at the net was a key factor for successful performance, where players could win more points but also tended to make less unforced errors.

The serve in tennis has been shown to be advantageous (Kovalchik and Reid, 2018), e.g., players win 67.3% of points with the 1st serve on a slow court surface (Gillet et al., 2009), but that proportion is greater in men’s singles compered to women’s (O’Donoghue, 2001). This is probably due to the significantly higher ball speed achieved by male players, which was only evident on serve, but not in other groundstrokes (Reid et al., 2016). Furlong (1995) found that the serve was more effective in tennis doubles compared to singles, likely due to the smaller target area for the return of serve (ROS) due to the server partner covering the net. This also allows the server in doubles to serve from a wider position and hence slice the ball further outside the court. The accuracy of the serve has not been widely studied although Vial et al. (2019) suggested that the landing accuracy measures in badminton were inappropriate. Whilst badminton is quite different from most other racket sports, since all shots are volleyed, the point can be made that trajectory, speed and spin, along with where the ball lands, all contribute to the difficulty associated with making a good ROS. Martin et al. (2019) analyzed first and second serves during 50 main draw 5 set matches during the 2014 Grand slam tennis events. They found fairly consistent ball velocities, percentage serves in and percentage points won between each of the five sets suggesting that professional tennis players can maintain serving performance over the course of a match. Whilst the authors suggested that small differences in serve velocity in the fifth set between match winners and losers may have led to the match outcome no other factors, such as trajectory and spin, were considered. Another factor not considered in this study is the ability to anticipate the serve trajectory, clearly if players can improve this ability during a match their ROS performance would potentially improve. Gillet et al. (2009) analyzed 116 matches, all lasted over 100 points, from the 2016 and 2017 French Open tournaments, to determine that flat serves to the T (centre of the court) and ROS to a central zone were the most effective in men’s singles tennis. This study recognised that multiple factors contribute to the effectiveness of both the serve and ROS and highlighted the fact that the coupling of these two “most important shots” leads to different strategies employed for both. Gómez et al. (2017) suggested that psychological factors, such as confidence and momentum, are likely to affect performance during a match, accounting for their finding that table tennis serving performance tended to fluctuate throughout the 140 men’s and women’s matches analyzed from the 2016 Olympic games.

O’Donoghue and Brown (2008) investigated the effectiveness of tennis serves showing that men’s first serves still had an impact on rally outcome in rallies that lasted four shots, i.e., servers won statistically more of these rallies. However, for second serves this advantage had diminished by the third shot. In table tennis, Zhang et al. (2013) assessed a player’s technique effectiveness using the “three phase evaluation theory.” This methodology calculated rally success rates for a player for rallies that lasted four shots or less, i.e., separate calculations for when serving and receiving, and for rallies that lasted over four shots irrespective of serving or not. The authors suggested players accorded high validity to these measures, particularly elite Chinese players. These studies suggest that the influence of the service extends some way into the rally in both tennis and table tennis although the service in both these sports are intuitively strong shots due to the speed in tennis and spin in table tennis. The impact of the serve in tennis was further corroborated by Fitzpatrick et al. (2019) who found that the player who won the most rallies containing 1 to 4 shots won the match almost 9 out of 10 times. This was the best predictor of match outcome from a range of measures which was somewhat surprising in that these matches were from the 2016 and 2017 French Open tournaments, played on the slowest surface, and where rallies were shown to be significantly longer than any of the other Grand Slams (O’Donoghue and Ingram, 2001).

The service in Padel is different to its closest similarity, the tennis service, because the rules dictate that it must be an underhand shot from a bouncing ball hit from below waist level. Thus, the ball cannot be hit as hard as in tennis although spin and the side wall can influence the difficulty in returning the shot. Additionally, similar to tennis doubles, the serving pair initially has an attacking opportunity because of the spatial advantage at shot three, i.e., one player at the net and the other approaching the net. This implies that the receiver is under some pressure to play an accurate ROS to try to prevent the serving pair from attacking by hitting the ball (shot 3) into a tactically advantageous area. This contention is supported by Courel-Ibáñez et al. (2014) who found that World padel tour players (*n* = 15 matches) won 83.4% of their service games. Ramón-Llin et al. (2013) found that, independent of performance level, the server in Padel, covered a significantly greater distance than his partner during rallies. To some extent this is obvious since the server immediately runs to the net following the service whereas the partner is standing at the net waiting for the ROS. However, lob returns over the serve partner’s head would probably negate this effect although Courel-Ibáñez et al. (2017) suggested that lobs tended to be directed to both sides of the court, near the walls but accounted for <16% of total shots played. This paper did not differentiate serve and ROS from other shots but did analyze spatial positioning, shot type and their effectiveness although only four players were analyzed during an unspecified number of matches.

At present little is known about the relationship between the serve and ROS, in all racket sports, other than the fact that the server tends to maintain the tactical advantage until around shot 5 when the advantage has dissipated. This knowledge has been gained from simple analyses of rally outcomes (e.g., O’Donoghue and Brown, 2008) or from experiential knowledge gained through exposure to elite match play (e.g., Zhang et al., 2013). Whilst the importance of a good ROS (technically and tactically) is well understood by coaches and players, there is little research to illustrate this. Zhang and Zhou (2017) differentiated specific serve tactics in table tennis that were associated with higher scoring rates whilst Vernon et al. (2018) qualitatively interviewed eight former or current top 250 professional male tennis players to reveal three types of returner. “Aggressive” returns put pressure on the server, “counter-punchers” got every return into court and the “neutral” played each serve according to its merits. This research also highlighted the difference between first and second serves in term of how aggressive the ROS could be. The objective of this research was to initially assess the effectiveness of the serve, in terms of winning the point, before analyzing quantitative data in relation to the effectiveness of the ROS and assess its impact on rally outcome. Since the ROS only directly impacts the third shot of the rally we decided to undertake an in-depth analysis of the ROS and third shots only. Serve type (first or second serve), service formation (Australian or Conventional), return type (flat or lob), see Table 1 for operational definitions, and playing standard were selected as parameters that could potentially affect the time, distance and velocity values of interest. Hence, the effectiveness of the ROS was analyzed using physical parameters of the serving pair at the time of the third shot being played to see whether these determined rally length and outcome.

**Table 1:** Operational definitions for Padel terminology used.

	Variations	Definition
Serve	1st or 2nd serve	The serve is hit from lower than the server’s waist after the ball bounces behind the serve line (Figure 1). The shot is usually played with back spin most often toward the opposite side wall.
Service formation	Conventional or Australian	At serve, the serve partner stands close to the net either on the other side of the court to the server (conventional) or always stays on the same side of the court (Australian) irrespective of the side where the serve takes place.
Flat return of serve (flat ROS)	Directed toward server, serve partner or between them	Service return is hit low over the net after the ball bounces. The direction is often determined by the serve formation and direction of serve.
Lob return of serve (lob ROS)	Directed toward server or serve partner	Service return is hit high over the net toward the back of the court.

The methodology used in this paper led to a couple of hypotheses. First, we thought that the serve would cease to have an effect on rally outcome after more than the four shots found for tennis singles (O’Donoghue and Brown, 2008), due to the territorial advantage gained by serving in doubles. Secondly, we hypothesised that if the ROS was effective the rally would tend to be longer and the winner of the rally would be unpredictable.

## Materials and Methods

### Participants

Matches took place at two tournaments, sanctioned by the Valencian Federation, in 2012 with 2000aaa prize money for each. All matches were processed using Tracker software, a newer version of the SAGIT/Squash software (Perš et al., 2008). The main sample consisted of 26 National (professionals playing on Pro tour) players (mean age 33.5 years, *SD* = 6.8) who played in 9 matches of the main draws. A further comparison sample of 30 Regional (elite amateur) players (mean age 31.1 years, *SD* = 6.9) who played in 9 matches during the qualification rounds was also used. The written consent of the tournament organisers was obtained to film and analyze the matches. All the participants signed an informed consent of their participation, which guaranteed the anonymity and exclusive use of the video recordings for scientific purposes. The Ethics Committee of University of Valencia (protocol H1494417717437) approved this study.

### Procedure

**FIGURE 1:**
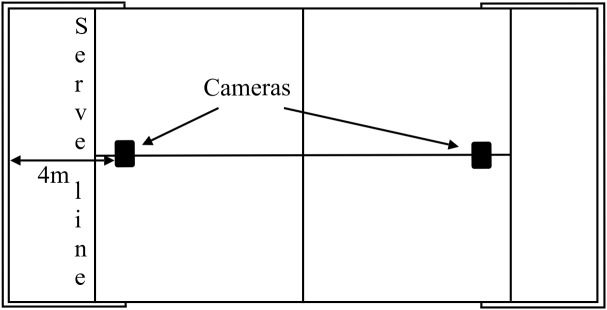
Placement of the cameras at the ceiling above the court.

Two digital Bosch Dinion Model IP 455 video cameras (Bosch, Munich, Germany) were used to film the matches (25 frames per second), sagittally placed over the courts at 6 m from the centre and over the service line (Figure 1).

The techniques for transferring video images into Tracker were identical to SAGIT/Squash, i.e., automatic processing with operator supervision, and have been well documented (Vučković et al., 2009). Similarly, the reliability for resultant calculations of distance and speed for each player (Vučković et al., 2010) and positions on court (Vučković et al., 2009) have been published.

### Data Processing

The shot type of the ROS (flat and lob) and x and y coordinates of the ball and player locations for each shot were recorded. James et al. (2007) suggested that reliability tests should reflect the way in which notation data is analyzed. Reliability measures for the Tracker software has been shown to be acceptable for analysis purposes (Vučković et al., 2010). Further measures were calculated for distinguishing a random sample of flat and lob ROS (*n* = 364) using both inter-operator (98.67% agreement, Kappa = 0.98) and intra-operator tests (99.33% agreement, Kappa = 0.99). Additional information regarding time between ROS and third shot, average speed of movement and distance covered for server between ROS and third shot and distance from the net for server and his partner at the third shot were recorded. Independent variables used to assess for ROS differences were (1) variables under the control of the serving pair which determine ROS difficulty, i.e., first or second serve and serve formation (conventional or Australian); (2) playing standard (National or Regional); (3) an outcome variable of serve effectiveness, rally length (short rallies of 7 shots or less or long). Finally, for lob ROS, whether the shot was directed at the server or serve partner was recorded.

To determine whether the service had a significant effect on the outcome of the rally Wilcoxon Signed Ranks tests and associated effect sizes (z score converted into effect size with >0.3 deemed medium and >0.5 large; Rosenthal, 1991) determined whether the serving pair won more rallies than receivers for rallies of different length. Friedman tests assessed whether the rally length had a significant effect on National and Regional players in similar ways.

Cluster analysis is a data mining technique that enables the formation of groups within a data set based on maximising the homogeneity of cases within a group and the heterogeneity between clusters (Hair et al., 1995). Cluster analysis begins with all cases as separate groups and the two “most alike” cases combined in the first step using the most appropriate distance measure. The two cases with the smallest distance measure will then cluster together and a group mean (cluster centroid) calculated and used in the next step. The next two most alike cases (or groups once cases have been clustered) are then combined. This process continues until an optimal cluster solution is obtained, based on the Silhouette coefficient, a measure of cohesion and separation. The number of clusters may also be changed if the optimal number of clusters is deemed practically not the best (Hair et al., 1995). In this study, the optimal number of clusters was deemed acceptable for both flat ROS (coefficient = 0.35, fair) and lob ROS (coefficient = 0.4, fair).

The two-step cluster analysis, using a probability-based log-likelihood distance measure (IBM SPSS Statistics, v.24, Chicago, IL, United States) enabled the continuous (two distance parameters, time, and average speed) and four categorical (serve type, serve formation, return type, and playing level) variables to be used.

## Results

As rallies increased in length the advantage for the serving pair diminished significantly for both Nationally (N) and Regionally (R) ranked players to the point where rallies of five or more shots (about two thirds of rallies) were equally likely to be won by either pair (Table 2). Thus, the serving pair only won more rallies of length 1 or 2 shots (large effect size) and 3 or 4 shots (medium effect size).

**Table 2:** Percentage of rallies won per match by serving pair in rallies of different numbers of shots (proportion of total rallies).

Playing	Rallies of 1	Rallies of 3	Rallies of 5	Rallies of 7	Rallies of	Friedman
standard	or 2 shots	or 4 shots	or 6 shots	or 8 shots	9+ shots	test χ^2^_5_
National	97.9 ± 4.2*ES	64.5 ± 5.0*ES	63.7 ± 12.1*ES	56.7 ± 14.7*ES	41.6 ± 9.6ES	16.2, *p* < 0.001
	0.96 (2.6%)	0.30 (11.5%)	0.27 (14.9%)	0.12 (12.8%)	0.16 (58.2%)	
Regional	94.9 ± 10.2*ES	65.8 ± 14.4*ES	56.8 ± 11.7*ES	51.6 ± 19.2 ES	46.8 ± 9.3	20.2, *p* < 0.001
	0.92 (2.3%)	0.36 (9.4%)	0.11 (10.2%)	0.09 (11.5%)	ES 0.06 (66.7%)	


Key: *Wilcoxon Signed Ranks test revealed serving pair won more rallies than receivers. ES, Effect size.

Return of serves were either hit flat (70.6%) or lobbed high toward the back of the court (29.4%). Flat shots were predominately directed at the server (71.9%) whereas lobs could be directed at either server (48.8%) or serve partner (51.2%). Flat shots, aimed at the serve partner, did not require this player to move very much to hit a shot, as already positioned at net for service, these returns were therefore excluded from the analysis.

26.8% of N level player’s ROS (cluster 5; Table 3) consisted of flat shots played against a first serve in a conventional formation (not Australian) compared to 29.5% of R player’s ROS (cluster 2; Table 3). The higher playing standard players tended to allow the server less time to play the shot (*N* mean = 0.86 s *SD* = 0.19 s; *R* mean = 0.98 s *SD* = 0.29 s) which occurred closer to the net (*N* mean = 3.84 m *SD* = 0.91 m; *R* mean = 4.46 m *SD* = 0.91 m) as the player’s average speed of movement was higher (*N* mean = 1.98 m/s *SD* = 0.47 m/s; *R* mean = 1.68 m/s *SD* = 0.56 m/s; Table 3). All of these rallies lasted more than five shots.

**Table 3:** Frequency of different rally characteristics and summary statistics of parameters related to the server hitting the third shot in each cluster for a flat return of serve (ROS).

										Time (between		Distance covered by				Average velocity of	
										ROS and		server (between ROS		Distance of server from		server (between ROS	
	Serve		Playing standard		Service formation		Rally length			third shot)		and third shot)		net (at third shot)		and third shot)	
							Short	Short		Mean	SD	Mean	SD	Mean	SD	Mean	SD
Cluster	1st	2nd	National	Regional	Australian	Conventional	(lost)	(won)	Long	(s)	(s)	(m)	(m)	(m)	(m)	(m/s)	(m/s)
1	0	178	95	83	94	84	17	27	134	0.90	0.24	1.64	0.55	4.13	0.87	1.88	0.53
	0.0%	90.8%	18.3%	16.6%	19.3%	15.7%	12.1%	14.5%	19.3%								
2	148	0	0	148	0	148	0	0	148	0.98	0.29	1.62	0.64	4.46	0.91	1.68	0.56
	18.0%	0.0%	0.0%	29.5%	0.0%	27.7%	0.0%	0.0%	21.4%								
3	257	0	135	122	122	135	114	143	0	0.92	0.25	1.67	0.56	4.12	0.88	1.89	0.53
	31.2%	0.0%	26.0%	24.4%	25.1%	25.3%	80.9%	76.9%	0.0%								
4	131	0	0	131	131	0	0	0	131	0.99	0.30	1.78	0.68	4.45	0.84	1.85	0.61
	15.9%	0.0%	0.0%	26.1%	27.0%	0.0%	0.0%	0.0%	18.9%								
5	139	0	139	0	0	139	0	0	139	0.86	0.19	1.65	0.49	3.84	0.91	1.98	0.47
	16.9%	0.0%	26.8%	0.0%	0.0%	26.0%	0.0%	0.0%	20.1%								
6	122	0	122	0	122	0	0	0	122	0.88	0.23	1.73	0.42	3.86	0.82	2.03	0.47
	14.8%	0.0%	23.5%	0.0%	25.1%	0.0%	0.0%	0.0%	17.6%								


*Key: Percentages calculated for columns but do not add to 100% because outliers were not included.*

Similarly, when N level players played a flat return of serve against an Australian formation first serve (23.5% of shots, cluster 6; Regional 26.1% of shots, cluster 4; Table 3) the N level players tended to allow the server less time to play the shot (*N* mean = 0.88 s *SD* = 0.23 s; *R* mean = 0.99 s *SD* = 0.30 s) which occurred closer to the net (*N* mean = 3.86 m *SD* = 0.82 m; *R* mean = 4.45 m *SD* = 0.84 m) as the player’s average speed of movement was higher (*N* mean = 2.03 m/s *SD* = 0.47 m/s; *R* mean = 1.85 m/s *SD* = 0.61 m/s). All of these rallies lasted more than five shots.

The spatial variables associated with the flat ROS off a 2nd serve (Cluster 1; Table 3) exhibited similar values to those for a flat ROS off 1st serves, with the exception of 9.2% of shots deemed outliers, and hence were not differentiated for any individual situation. Finally, cluster 3 (ROS off 1st serve) occurred only in rallies that ended within four shots, although either pair could have won the rally (Table 3) and none of the four spatial variables were unusual.

When National players played lob ROS they usually achieved similar outcomes, irrespective of which opponent they played the shot to, or whether returning a first [opponent distance to net (mean = 5.43 m *SD* = 1.67 m) for the 66.5% of shots in cluster 1; Table 4] or second serve [opponent distance to net (mean = 5.57 m *SD* = 1.07 m) for the 14.4% of shots in cluster 2; Table 4]. These returns resulted in long rallies 48.8% [63/(44+22+63), cluster 1, Table 4] and 61.8% [63/(22+17+63), cluster 2, Table 4], respectively.

**Table 4:** Frequency of different rally characteristics and summary statistics of parameters related to either opponent hitting the third shot in each cluster for lob return of serves (ROS).

												Distance covered by opponent		Distance of by opponent	
					Player ROS					Time (between ROS		hitting third shot (between		hitting third shot from	
	Serve		Playing standard		aimed toward		Rally length			and third shot)		ROS and third shot)		net (at third shot)	
						Serve	Short	Short		Mean	SD	Mean	SD	Mean	SD
Cluster	1st	2nd	National	Regional	Server	partner	(lost)	(won)	Long	(s)	(s)	(m)	(m)	(m)	(m)
1	129	0	129	0	62	67	44	22	63	1.65	0.48	2.68	1.19	5.43	1.67
	26.0%	0.0%	66.5%	0.0%	21.1%	20.1%	28.8%	21.4%	17.0%						
2	0	102	28	74	43	59	22	17	63	1.65	0.23	2.43	0.79	5.57	1.04
	0.0%	78.5%	14.4%	17.1%	14.6%	17.7%	14.4%	16.5%	17.0%						
3	126	0	0	126	55	71	83	43	0	1.64	0.42	2.34	1.03	5.03	1.56
	25.4%	0.0%	0.0%	29.1%	18.7%	21.3%	54.2%	41.7%	0.0%						
4	80	0	0	80	0	80	0	0	80	1.69	0.23	2.67	0.90	5.46	1.05
	16.1%	0.0%	0.0%	18.5%	0.0%	24.0%	0.0%	0.0%	21.6%						
5	74	25	32	67	47	52	3	17	79	3.03	0.28	5.82	0.86	8.61	0.37
	14.9%	19.2%	16.5%	15.5%	16.0%	15.6%	2.0%	16.5%	21.3%						
6	86	0	0	86	86	0	0	0	86	1.72	0.26	2.42	0.83	5.96	0.92
	17.3%	0.0%	0.0%	19.9%	29.3%	0.0%	0.0%	0.0%	23.2%						


*Key: Percentages calculated for columns but do not add to 100% because outliers were not included.*

The rest of the lob returns (16.5% cluster 5; Table 4), and occurred in all situations, resulted in the opponent hitting the ball moving greater distances (mean = 5.82 m *SD* = 0.86 m) to positions further from the net (mean = 8.61 m *SD* = 0.37 m) and over a great time (mean = 3.03 s *SD* = 0.28 s) than the other lob returns and 79.80% [79/(3+17+79)] of the time resulted in long rallies.

Regional player’s lob returns also achieved clusters 2 (17.1%) and 5 (15.5%; Table 4). Regional player’s lob shots achieved slightly different results when played to the server (distance to net: mean = 5.96 m *SD* = 0.92 m and distance opponent moved: mean = 2.42 m *SD* = 0.83 m) compared to the serve partner (distance to net: mean = 5.46 m *SD* = 1.05 m and distance opponent moved: mean = 2.67 m *SD* = 0.90 m) when returning first serves that resulted in long rallies. However, 29.1% of Regional player’s lob ROS off first serves (cluster 3) always resulted in short rallies {winning 34.9% [43/(83+43)] of them} where the opponent’s distance to the net (mean = 5.03 m *SD* = 1.56 m) and distance covered (mean = 2.34 m *SD* = 1.03 m) tended to be lower than any other cluster.

## Discussion

The serving pair for Padel players were found to have a significant advantage in rallies, which lasted until shot 8 for National and shot 6 for Regional level players. However, effect sizes clarified that this advantage diminished once the rallies lasted over four shots although National level players appeared to maintain an advantage closer to a medium effect (0.27) for rallies of five or six shots. This suggests that the serve advantage is greater for better players due to them being more able to play winners, or force errors from their opponents, from an advantage situation. This finding probably reflects the nature of Padel in that it is much harder to play a winner, due to the court dimensions and structure, meaning that when a pair is dominating a rally, as for the start of the rally when serving, it often takes more shots to finish the rally compared to tennis. Hence, the defending pair is more likely to be able to stay in the rally, i.e., the defending team is able to return more shots, shown to be determined by level of skill in this study, and therefore also more likely to return the rally to a more equal situation than evident in tennis. This would suggest that rallies are longer in Padel than tennis, backed up by previous studies which found the average rally to be 15 s for professional Padel players (Almonacid, 2012). The service in tennis has been shown to be advantageous in many studies (e.g., Kovalchik and Reid, 2018). Tennis serves tend to increase the probability of winning the rally, but the extent of this advantage is determined by court surface (O’Donoghue and Ingram, 2001) and length of rally, with the effect of the serve seemingly having dissipated by the third or fourth shot (O’Donoghue and Brown, 2008). In tennis, the advantage is based on speed of serve, direction and court size. Padel, uses different equipment and playing area, is therefore likely to exhibit differences in terms of serve advantage, as well as for other factors.

In the ROS players hit about 70% flat and 30% lob shots potentially reflecting the need for the server to run toward the net and hence leave enough space at the net to make the flat ROS more advantageous. The fact that over 70% of flat ROS was directed at the server supports this view. Courel-Ibáñez et al. (2015) showed how important playing at the net was to winning rallies in Padel. In tennis, Gillet et al. (2009) found a relationship between the effectiveness of the ROS and the direction of the serve. This study did not consider serve direction, which may have impacted on the ROS decision. Torres-Luque et al. (2015) found that about 75% of serves were directed to the backhand and this should therefore be included in future studies.

However, two thirds of flat ROS resulted in long rallies (8 or more shots) with National level players achieving this on 65.9% of their flat ROS against first serves. Comparing National and Regional players on flat ROS, the time, distance and speed parameters showed that National players hit the ball harder than Regional ones. Previously, Courel-Ibáñez et al. (2015) suggested that the best players should be aggressive when returning and adopt a defensive style when serving. Similarly, Vernon et al. (2018) differentiated “aggressive” and “neutral” ROS in tennis. However, in response to the ROS, National players approached the net faster than Regional players and were thus able to hit the ball nearer the net, and hence being in a strategically advantageous position (Courel-Ibáñez et al., 2015). This was accomplished in both conventional and Australian formations even though the task demands for the server were different, player had to move quicker due to further distance, as suggested by Ramón-Llin et al. (2013).

When using a lob ROS, players did not favor hitting to one player over the other, corroborating the findings of Almonacid (2012) who found that professional Padel players hit equally to the deuce and advantage sides. The key objective for the lob was obviously depth, as short lobs present an easy opportunity for a powerful smash or tray. National players’ lob ROS tended to either achieve good (around 5.5 m beyond the net) or excellent (around 8.5 m) depth. Decisively, these lob ROS achieved long rallies 48.8% of the time for good depth off first serves, 61.8% of the time for good depth off second serves and 79.8% of the time for excellent depth irrespective of serve. For Regional players, however, if their lob ROS achieved a depth of about 5.5 m or more they always achieved long rallies whereas if they only achieved 5.0 m depth the rallies were always short. It would seem, therefore, that Regional players were still playing fairly weak lob ROS on occasion, something very unusual at National level. Another perspective related to the lob ROS is to consider it as an attacking shot. Indeed, Muñoz et al. (2017) presented how the best way to move from defense to attack was to gain the net using the lob. Taking these into consideration it seems clear that National level players were more able to consistently negate the offensive nature of the serve using accurate lobs whereas Regional players were less successful due to the presence (29.1%) of lobs that lacked sufficient depth.

Whilst this study assessed detailed parameters associated with the outcome of the ROS some parameters associated with the serve were not included in the analyses. The time between serve and ROS and the direction of the serve were not included even though Vial et al. (2019) reported that speed and trajectory impacted on the difficulty of the ROS. However, the serve in Padel has been shown in this paper to exert different pressure on the opponents than in tennis. Whilst it is common to assume a significant difference between first and second serves (consistently shown in tennis, e.g., O’Donoghue and Brown, 2008) cluster analysis did not indicate significant differences for the distance, time and speed parameters associated with a flat ROS in Padel. This may be due to the relative ease of playing a good service in Padel but the difficulty in playing a very good one. Since the rules dictate an underhand service it would seem that hitting unreturnable serves are very unlikely and even forcing very weak ROS unlikely, less than 15% of National rallies ended before five shots had been played. However, future studies should consider other variables in relation to the serve and ROS including the time between the two and some consideration should be given to classifying attacking and defending positions since this distinction appears important for determining shot selections. It would be reasonable to analyze these parameters during and between all shots and hence using more in-depth quantitative analysis to determine serve and ROS impact on rally outcome. Finally, since playing standard has a clear impact on performance, future studies need to sample the best players with respect to the parameters studies here.

## Practical Applications

The findings of this study suggest that coaches should consider teaching return of serve shots from a tactical perspective. Given that in short rallies, up to around 6 to 8 shots depending on skill level, the server has a significant advantage, the aim of the ROS is to prevent the serving pair winning the rally quickly. This is best achieved by good depth on lobs, regardless of the direction, and pace on low shots, predominately aimed toward the server.

## Data Availability

All datasets generated for this study are included in the manuscript and/or the supplementary files.

## Ethics Statement

This study was approved by the Ethics Committee of University of Valencia (protocol H1494417717437).

## Author Contributions

JR-L, RM-G, JG, and SL undertook data acquisition and processing. JR-L, NJ, and GV designed the study, conducted the analysis, interpreted the data, and wrote the manuscript. All authors read and approved the final manuscript.

## Conflict of Interest Statement

The authors declare that the research was conducted in the absence of any commercial or financial relationships that could be construed as a potential conflict of interest.
